# Understanding Kenyan policymakers’ perspectives about the introduction of new maternal vaccines

**DOI:** 10.1093/heapol/czae059

**Published:** 2024-07-02

**Authors:** Rupali J Limaye, Berhaun Fesshaye, Prachi Singh, Rose Jalang’o, Rosemary Njura Njogu, Emily Miller, Jessica Schue, Molly Sauer, Clarice Lee, Ruth A Karron

**Affiliations:** Department of International Health, Johns Hopkins Bloomberg School of Public Health, 615 N Wolfe Street, Baltimore, MD 21205, United States; Department of International Health, Johns Hopkins Bloomberg School of Public Health, 415 N Washington Street, Baltimore, MD 21205, United States; Department of International Health, Johns Hopkins Bloomberg School of Public Health, 415 N Washington Street, Baltimore, MD 21205, United States; National Vaccines and Immunization Program, Ministry of Health Kenya, Afya House, Cathedral Road, P. O. Box 43319–00100, Nairobi, Kenya; Jhpiego Kenya, 2nd Floor, Arlington Block, 12 Riverside, off Riverside Drive, P.O. Box 66119-00800, Nairobi, Kenya; Department of International Health, Johns Hopkins Bloomberg School of Public Health, 615 N Wolfe Street, Baltimore, MD 21205, United States; Department of International Health, Johns Hopkins Bloomberg School of Public Health, 615 N Wolfe Street, Baltimore, MD 21205, United States; Department of International Health, Johns Hopkins Bloomberg School of Public Health, 615 N Wolfe Street, Baltimore, MD 21205, United States; Department of International Health, Johns Hopkins Bloomberg School of Public Health, 615 N Wolfe Street, Baltimore, MD 21205, United States; Department of International Health, Johns Hopkins Bloomberg School of Public Health, 615 N Wolfe Street, Baltimore, MD 21205, United States

**Keywords:** Policy, maternal and child health, immunization, policy process

## Abstract

New vaccine policy adoption is a complex process, especially in low-and-middle-income countries, requiring country policymakers to navigate challenges such as competing priorities, human and financial resource constraints, and limited logistical capacity. Since the beginning of the Expanded Programme on Immunization, most new vaccine introductions under this structure have not been aimed at adult populations. The majority of adult vaccines offered under the Expanded Programme on Immunization are not typically tested among and tailored for pregnant persons, except those that are specifically recommended for pregnancy. Given that new maternal vaccines, including respiratory syncytial virus and group B streptococcus vaccines, are on the horizon, it is important to understand what barriers may arise during the policy development and vaccine introduction process. In this study, we sought to understand information needs among maternal immunization policymakers and decision-makers in Kenya for new vaccine maternal policy adoption through in-depth interviews with 20 participants in Nakuru and Mombasa, counties in Kenya. Results were mapped to an adapted version of an established framework focused on new vaccine introduction in low-and-middle-income countries. Participants reported that the policy process for new maternal vaccine introduction requires substantial evidence as well as coordination among diverse stakeholders. Importantly, our findings suggest that the process for new maternal vaccines does not end with the adoption of a new policy, as intended recipients and various actors can determine the success of a vaccine programme. Previous shortcomings, in Kenya, and globally during human papillomavirus vaccine introduction, show the need to allocate adequate resources in education of communities given the sensitive target group. With maternal vaccines targeting a sensitive group—pregnant persons—in the pipeline, it is an opportune time to understand how to ensure successful vaccine introduction with optimal acceptance and uptake, while also addressing vaccine hesitancy to increase population benefit.

Key Messages

- Participants reported that the policy process for new maternal vaccine introduction requires substantial evidence as well as coordination among diverse stakeholders.
- Processes to introduce new maternal vaccines do not end with the adoption of a new policy, as vaccine recipients and relevant stakeholders must be educated and willing to accept the vaccine through community sensitization and stakeholder engagement.
- With the introduction of new maternal respiratory syncytial virus and group B streptococcus vaccines in Kenya targeting pregnant people, a group outside of the traditional Expanded Programme on Immunization realm, ensuring acceptance and uptake is key for disease reduction.

## Introduction

Since the inception of the World Health Organization (WHO) Expanded Programme on Immunization (EPI) in 1974, countries have had to make decisions related to the vaccines included in their national immunization programmes. Today, 13 antigens are included in the WHO’s EPI for routine use, with other antigens recommended for specific geographies or higher-risk populations (Guignard *et al*., 2019; World Health Organization, 2023a). While vaccines have helped to greatly reduce morbidity and mortality against a range of diseases, many low- and middle-income countries are likely to fall short of immunization targets outlined in Sustainable Development Goal 3 (Guillaume *et al*., 2023). Country-level policymakers are faced with the challenge of identifying which antigens should be recommended for routine use, within the context of competing priorities, human and financial resource constraints, and limited logistical capacity (Guignard *et al*., 2019; Guillaume *et al*., 2023).

New vaccine policy adoption is complex, particularly in low- and middle-income countries settings. Global financing and implementation partners play a key role, and, at the country-level, policy adoption is guided by country-level policymakers and decision-makers, including National Immunization Technical Advisory Groups (NITAGs), EPI officials and other Ministries. Policymakers and decision-makers must consider the safety, efficacy and potential impact of a new vaccine with broader implementation factors, including health-system capacity (Guignard *et al*., 2019; Donadel *et al*., 2021; Guillaume *et al*., 2023). Furthermore, the EPI structure has primarily focused on vaccines for paediatric or adolescent populations; and countries face unique challenges related to the introduction of vaccines targeted to other populations like adults broadly or pregnant persons specifically (Guignard *et al*., 2019; Abdullahi *et al*., 2020). Besides limitations related to the EPI structure, it is critical that country-level policymakers and decision-makers are sufficiently prepared to make decisions related to the introduction and delivery of vaccines targeted for groups outside of paediatric or adolescent populations to optimize vaccine uptake (Gallagher *et al*., 2017; 2018; Guignard *et al*., 2019; Abdullahi *et al*., 2020; Donadel *et al*., 2021; Otieno *et al*., 2021). Inadequately preparing decision-makers, health systems and communities for vaccination outside of the traditional age groups can lead to delayed access and uptake.

One framework proposed in 2010 (Levine *et al*., 2010) focused on addressing potential issues and challenges in the new vaccine adoption process. This framework was created based on experiences with Haemophilus influenzae type b, pneumococcal and rotavirus vaccine adoption and places new vaccine adoption steps into a continuum from evidence to access in the hope of improving vaccine initiative efficiency (Levine *et al*., 2010). A few notable changes have occurred since the introduction of Levine *et al*.’s framework. New maternal vaccines in development are targeted and tested in pregnant individuals (Engmann *et al*., 2020). Pregnant individuals are usually excluded from vaccine clinical trials and many vaccines administered during pregnancy—tetanus, influenza and COVID-19 vaccines—are also given to other populations outside of pregnancy (Chu and Englund, 2014). Furthermore, vaccine hesitancy in low- and middle-income countries, and globally, has steadily increased over previous decades, landing it on the WHO’s list of top threats to global health (World Health Organization, 2019; Nuwarda *et al*., 2022). Vaccine hesitancy is a complex, multi-faceted issue that can result from misinformation about vaccine safety, cultural beliefs or lack of knowledge (Simas and Larson, 2021; Galagali *et al*., 2022; Nuwarda *et al*., 2022). Community engagement has been shown to be the most influential approach to decrease vaccine hesitancy, with communication needing to come from trusted individuals such as healthcare providers (Galagali *et al*., 2022; Mutombo *et al*., 2022; Nuwarda *et al*., 2022).

Kenya, the setting of the current research, shifted to a decentralized system of governance in 2013 with the creation of 47 semi-autonomous counties (McCollum *et al*., 2018; Masaba *et al*., 2020). Commonly known as devolution, this transfer of authority from national to subnational entities sought to strengthen community participation and reinforce democracy (McCollum *et al*., 2018). With devolution, planning, management and budgeting of health service delivery are left in the control of the counties while health policy creation is left to the national government (Masaba *et al*., 2020; Gichuki, 2021). Vaccine policy creation follows this structure, with formulation occurring at the national level and policies being transferred to counties for implementation (Otieno *et al*., 2021). However, unclear role divisions between national and county level entities have been reported and have hindered policy implementation in the past (Otieno *et al*., 2021). In Tanzania, another country with a decentralized government, positive outcomes have occurred, such as increased autonomy in subnational resource mobilization and utilization, as well as challenges such as insufficient community participation (Frumence *et al*., 2013). In Ghana and Malawi, district-level decision-makers have noted that despite decentralization, the national Ministry of Health still holds influence on district health-system decisions, highlighting an important power dynamic (Bulthuis *et al*., 2021).

Several vaccines specifically designed for use in pregnancy are on the horizon—including respiratory syncytial virus (RSV) vaccines and group B streptococcus (GBS) vaccines—and countries will have to make decisions about whether or not to include these new vaccines in their national immunization programmes (Geoghegan *et al*., 2022). Proactively preparing policymakers and decision-makers and identifying potential barriers for acceptance and delivery of future vaccines during pregnancy will be key to realizing their individual- and population-level benefits (Otieno *et al*., 2021).

Understanding how policymakers and decision-makers consider and develop new vaccine introduction policies, including those targeting non-traditional target populations, is crucial for successful adoption and implementation of new maternal vaccines. Specifically in settings like Kenya, understanding the experiences and attitudes of subnational officials leading policy implementation is vital. In this study, we used an adapted vaccine policy framework to understand information needs among relevant policymakers and decision-makers in Kenya for uptake of future maternal vaccines.

## Methods

In-depth interviews were conducted with local policymakers and decision-makers. Participants were recruited from two counties, with two communities in each county: Nakuru (rural) and Mombasa (urban). We chose Kenya because it is one of the countries that may provide maternal vaccines soon after licencing and has strong political will related to the development and implementation of maternal vaccination policies. In consultation with in-country stakeholders including the Ministry of Health and other vaccine-relevant bodies, Nakuru and Mombasa counties were selected, given the diverse populations they serve. Potential participants were identified in consultation with local county government officials working in immunization in Kenya. These consultations resulted in a list of organizations and potential participants also working in maternal immunization for recruitment. For this study, we defined policymakers and decision-makers as those that play a role in immunization policy in the country. For Kenya, this included members of the national immunization technical advisory group (KENITAG), Kenya Obstetrical and Gynaecological Society (KOGS), Kenya Paediatric Association (KPA), National Nurses Association of Kenya, and at county level we interviewed county health directors, and public health, reproductive health and immunization coordinators. The research assistants contacted participants through email and phone.

Data were collected in August and September 2022. Data collectors participated in a 3-day training exercise and completed an online human ethics training course. If a participant was interested in joining the study, a research assistant then set up a time via phone or in person to explain the purpose of the study and participant eligibility was ascertained. If a participant met the inclusion criteria (at least 18 years of age, able to give consent and agreed to participate), oral consent was obtained. Interviews were primarily conducted in English, but Swahili and other local languages were used as necessary. All interviews occurred in person in a semi-private setting. All interviews were audio recorded, transcribed and translated into English by external translators fluent in both languages. All data, including audio recordings, were stored on encrypted servers, and only members of the study team had access to the data.

Half of the participants were interviewed primarily about RSV disease and vaccines while the other half were interviewed about GBS disease and vaccines. However, all interviews included discussions about general maternal vaccine policy and results presented are based on such discussions. General policy questions included ‘As far as you know, how are new vaccines adopted in your country/community? Can you describe the process’ and ‘As far as you know, what are the factors that influence new vaccine policies in your country/community?’

A team of six analysed the data using Atlas.ti for data management. The code list was developed, refined and finalized over three rounds of open coding. Following agreement of a code list, the transcripts were then coded, with the team holding discussions on emerging themes after coding 50% of the transcripts. Two members of the team conducted inter-rater reliability with ∼10% of the transcripts that neither of them had coded (three transcripts). Reliability was 89%. The team then identified themes and sub-themes. This study received ethical approval from the Kenya Medical Research Institute and the Johns Hopkins Bloomberg School of Public Health.

We used grounded theory during data analysis and allowed themes to emerge from the data without a framework identified at the onset of analysis. Once data analysis was complete, we looked at existing vaccine policy frameworks that included domains that aligned with our results. Based on our emergent themes, we found that our results could be organized using the Levine *et al*. (2010) framework that outlines steps in accelerating the adoption of new vaccines (see Figure 1a). The Levine *et al*. (2010) framework, as previously described, is one way to organize the policy introduction process. We have adapted this framework based on our results, adding community sensitization and stakeholder training to the figure (See Figure 1b). Both the original and adapted frameworks include three key steps: (1) establish and organize evidence; (2) establish supportive global policies; and (3) translate policies into local action. In the sections below, we synthesize findings from our interviews related to each of these steps.

**Figure 1.**
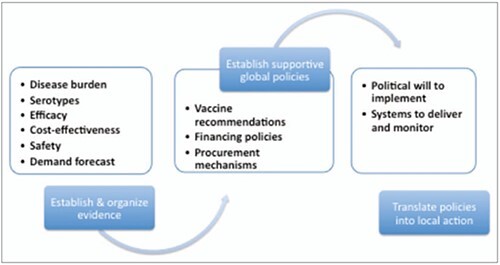
(a) Evidence to policy implementation for vaccine introduction framework Levine *et al*. (2010).

**Figure 1b.**
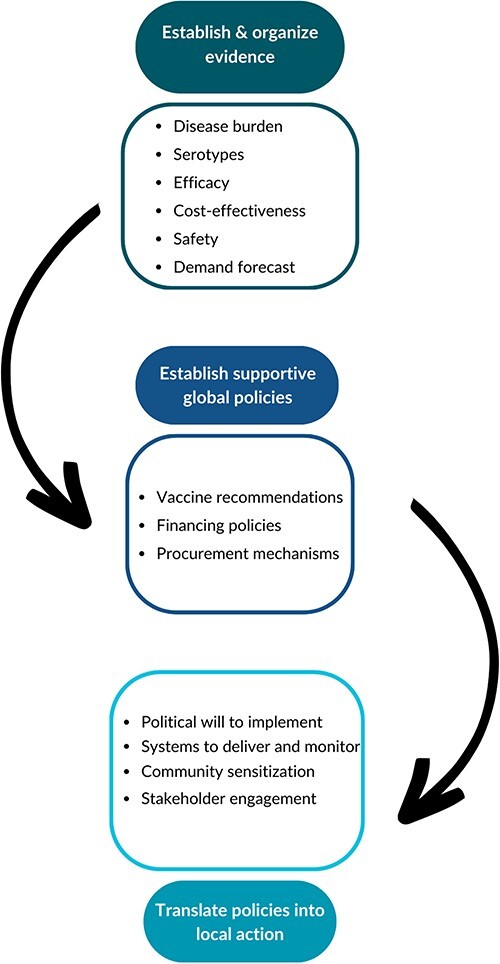
(b) Adapted Levine *et al*. (2010) framework

## Results

We interviewed 20 county-level policymakers and decision-makers, evenly split between Mombasa and Nakuru counties. Twelve participants identified as female, with the remaining eight identifying as male. Participant roles were as follows: six consultant physicians, five county-level directors, one promotion officer, four health service coordinators and four public health officers.

## Establish and organize evidence

The vaccine introduction process starts with establishing the epidemiology and burden of the target disease as well as the safety, efficacy and cost-effectiveness of the vaccine being introduced (Levine *et al*., 2010). Participants in our study noted a process similar to the Levine *et al*. (2010) framework in that the information needed before introduction of a new maternal vaccine includes disease burden, vaccine safety, vaccine efficacy, cost-effectiveness and recipient demand for the vaccine. Participants indicated that it was important for disease burden studies to be context-specific, as this policymaker from Mombasa stated:

‘*My understanding is that before the introductions of any new vaccines, there has to be studies that are done at the country level and once there has been evidence of the benefits to this vaccine, the efficacy, then through the divisions of vaccines and the immunizations they take up this and look at the whole country and see the vulnerability of the population*’ (interviewed about RSV, PM_09).

Another policymaker from Nakuru noted:

‘*We need to know how common the disease is. That means we have to make the right diagnosis and confirm that this is a problem in the society… Then you may ask yourself, what is the likelihood, now after you have confirmed that this baby is likely to die or have lung chronic disease… So even when you are telling the mother, that is the first question she asks you; how common this disease is?*’ (RSV, PM_05).

Participants emphasized the importance of vaccine safety, especially given that pregnant people are the target population for upcoming RSV and GBS maternal vaccines; as one policymaker from Mombasa stated:

‘*Given that a pregnant mother has another life inside her, that is a very sensitive target group, so it would be very important for all the national and county bodies to ensure that whatever they are bringing will not harm the mother or the fetus*’ (RSV, PM_10).

One policymaker from Nakuru discussed the importance of both safety and efficacy for not only those creating policy, but the general population as well:

‘*Because the general population knows other vaccines which are already in use have worked. So, once they hear of a new vaccine, they want to be convinced… safety has to be there and then the effectiveness definitely has to be there because, why should we introduce a substance which the efficacy may not make any impact in the first place?*’ (RSV, PM_01).

Cost-effectiveness of a new vaccine was also reported as an important aspect of new vaccine policy by this policymaker from Nakuru:

‘*The economic impact of the disease can also be a good selling point because when we talk with the policy makers and we ensure that we put the economic benefits of that vaccinating one to not vaccinating one, and the impact one will have on the healthcare system/economy*’ (RSV, PM_03).

## Establish supportive global policies

After technical evidence is established, the next step in the policy process is translation into actionable policies through collaboration between international policy bodies such as the WHO and domestic bodies such as NITAGs (Levine *et al*., 2010). When asked about the policy formulation process in Kenya, our participants noted that policy influences come from global organizations such as the WHO, which are then adopted after review by KENITAG. In the case of maternal and child vaccines, KOGS and KPA are also consulted. One policymaker from Nakuru stated:

‘*There is the Kenya Immunization Advisory Group, the KENITAG, they’re the ones who advise the Ministry whether to introduce a vaccine or not, whether it is necessary*’ (GBS, PM_01).

A policymaker from Mombasa noted that given the target audience of RSV and GBS vaccines, it was imperative to involve obstetricians and gynecologists’ input in recommendations:

‘*I think for a maternal vaccine, the Kenya Obstetric and Gynecological Society must also be involved because they are the ones who are going to advise because they would like to know whether their gynaecologists recommend this vaccine before they actually take it up. So, it would be very important to involve these professional bodies fully, even before we think about the introduction*’ (RSV, PM_09).

One participant believed that a recommendation from KOGS would positively affect uptake:

‘*Any new vaccine that is being introduced to the market, has gone through the different steps, has been approved, has been recommended by the national KOGS and given a greenlight, will be embraced by any woman, disregarding their religious alignment, education and economic status*’ (GBS, PM_07).

Also noted in this step is the importance of outlining financing and procurement processes, especially in Gavi, the Vaccine Alliance (Gavi) eligible countries, for policy success. Our participants reported the significance of possible financial obstacles and the need to proactively address them before new maternal vaccine introduction. As this policymaker from Mombasa stated:

‘*Then the issue of the cost comes in, is there a cost to the client and even the cost to the government because that is very important in terms of sustainability*’ (RSV, PM_08).

Another policymaker from Nakuru said:

‘*The largest, the biggest challenge is cost… we are dealing with a poor population, unfortunately you have to tell us whether the government will cater for that vaccine, for us to be able to implement it*’ (RSV, PM_05).

As for procurement mechanisms, participants discussed the role of organizations like UNICEF and Gavi, with a policymaker from Mombasa stating:

‘*UNICEF plays a very big role when it comes to vaccine supply. The WHO gives the recommendations, we pick from those recommendations and we adapt to our country… Gavi has been very supportive, most of our cold chain equipment was supported by Gavi*’ (RSV, PM_07).

## Translate policies into local actions

Once policies are formulated, concerted effort between bodies at all levels, including global, regional, national and sub-national must occur during implementation to ensure policies are put into place (Levine *et al*., 2010). Policies may go unimplemented if local officials were not involved in formulation or are unaware of the evidence backing a policy. This step’s importance mapped to our results as policymakers noted that policies are created at the national level and then disseminated to the county level for implementation, which points to the importance of political will for local implementation. As a policymaker from Mombasa noted:

‘*As a county we’ve not come up with our own policies on vaccines, so we’ve always adopted the national ones’* (GBS, PM_08).

Another policymaker from Nakuru echoed this by saying:

‘*Because most of the policies that we are implementing at the counties, the majority follow the adopted process where the recommendations from WHO are adopted within the national*’ (RSV, PM_04).

Once a policy is in place, participants discussed implementation of those policies to translate them into uptake. This policymaker from Nakuru suggested integration of new maternal vaccines into the antenatal care structure:

‘*For us to get the buy-in even for our women we will want to produce it using structures that we have. We don’t want to get a parallel system… You know if we integrate it well in the systems that we are having then I don’t think there will be much of a problem’* (RSV, PM_04).

Another policymaker from Mombasa also discussed the potential benefits of integration:

‘*Then the issue of whether it can be integrated with the other vaccines because already we are aware that there are several vaccines that are being administered all through to the infant and even to the mother. How easy is it to integrate this new vaccine with the existing vaccines? Is the schedule aligned to the others because they come for several visits, and they are targeted this is like through focus antenatal care. In most cases it is important that the vaccines are targeted towards these visits because anything outside this then there will be too many visits and that might reduce the acceptability of this vaccine’* (RSV, PM_08).

Two topics that were not explicitly mentioned by the Levine *et al*. (2010) framework emerged as important in this study, namely community sensitization and stakeholder engagement. Almost all participants noted the importance of educating the target audience, pregnant people, and influential individuals and entities around them. Simply having a policy in place for vaccine distribution to the target population may not guarantee uptake and reduction of disease burden, as this policymaker from Nakuru explained:

‘*One of the biggest challenges in adopting a new vaccine is resistance from the community level. People just refuse it, so resistance is the biggest challenge*’ (GBS, PM_02).

A policymaker from Mombasa stated:

‘*You have to consult the recipients before a new vaccine is introduced—the community members, sensitization of the community is very key and critical, they should clearly understand the benefits of this vaccination and once they actually understand and they appreciate the benefits of the vaccination, then it becomes easier for them to accept to be vaccinated*’ (RSV, PM_09).

Additionally, the same policymaker also emphasized the necessity of educating men specifically given their influence:

‘*The person who has the voice is actually the man in the family but unfortunately it is the women who attend to the facilities. So, without actually bringing on board the men so that they can actually accept that vaccination, then you might find a lot of resistance*’ (RSV, PM_09).

Another policymaker spoke on the importance of community engagement based on their previous experiences:

‘*All the programmes at the moment—we have the voice of the user at all levels, including peer to peer then it synergizes on the overall process*’ (RSV, PM_04).

This policymaker form Mombasa discussed the sensitivities around vaccinating pregnant people and the need for community engagement:

‘*Community participation is key, you know a pregnant woman is very sensitive and there has never been much apart from the tetanus toxoid (TT) which is known to protect the mother, you know the baby during the delivery, when you want to introduce vaccines to pregnant women… sensitize them and they should know the burden of this RSV*’ (RSV, PM_07).

Stakeholder outreach and training was also brought up by participants, with stakeholders including healthcare providers, religious and community leaders, and media being identified. Participants noted religious leaders as a central stakeholder given the history of vaccine hesitancy and because they hold the trust of their followers. As one policymaker from Mombasa stated:

‘*Bring all the religious leaders on board before implementing any vaccine because they are all involved, Hindus, Christians, Muslims they are leaders. I think there are quite a number of them who are anti-vaccines so… involve them, even those who are atheists*’ (RSV, PM_08).

This policymaker from Mombasa echoed the importance of religious leaders as well as other community leaders:

‘*During the campaigns we normally sensitize the community because if you do not, there are so many rumours, myths and misconceptions in terms of the vaccines. So, the best thing is just to educate the gatekeepers… we start with the gatekeepers, the chiefs, the imams the religious leaders, we sensitize them then after that we go to the other community liaison like the village elders, then the community health volunteers (CHVs) themselves’* (GBS, PM_07).

Healthcare providers were also cited as vital stakeholders during the policy implementation process due to their role of interacting directly with patients, as this policymaker from Nakuru describes:

‘*The decision makers [for adoption of new vaccines] are the healthcare providers, the main decision makers in the healthcare system are specialists. Specialists in this case are the consultants because those are the ones who disseminate information within the various departments. So, I think those are the key people, as long as they are convinced then they will convince their staff*’ (GBS, PM_06).

Media, including both traditional and social media, can also play a central role in the uptake of vaccines, as this policymaker from Mombasa notes:

‘*Media has a big role can also be used negatively or positively to actually promote or fail or increase the consumption of these vaccines; it depends on the information that is given out by the media*’ (GBS, PM_09).

A policymaker from Mombasa described the negative effect of lacking stakeholder engagement and community sensitization in the rollout of HPV vaccines:

‘*For instance the HPV when it was introduced, I can say that it was not done well… it was kind of done in a rush. We didn’t have a meeting with all the relevant stakeholders and we brought on board teachers, but as I said it was in a hurry so not everyone understood what it was meant for. We are experiencing difficulties in actually ensuring parents accept or even teachers trying to convince the parents that the HPV vaccine is necessary to the children or rather to the targeted audience. If there is a new vaccine… there is need for the stakeholders to be involved right at the beginning, there is need for information to be given so that we don’t end up with rumours*’ (RSV, PM_10).

## Discussion

Through in-depth interviews with county-level policymakers and decision-makers in Kenya, we found that the policy process for new maternal vaccine introduction, from formulation to implementation, requires substantial evidence of various types as well as coordination among diverse stakeholders. Participants noted the strong influence of the national government even under a decentralized system by reporting their involvement in policy implementation rather than policy creation. Additionally, to realize a vaccine’s benefits, the process does not end with the adoption of a new policy, as vaccine beneficiaries and relevant stakeholders must be educated and willing to accept the vaccine. Participants reported that new maternal vaccine introduction requires data on disease burden, vaccine safety, vaccine efficacy, cost-effectiveness and recipient demand for the vaccine. Suggestions for implementation of future maternal vaccines included integration into existing antenatal care structures for ease of administration and increased uptake. Finally, participants stressed the importance of community sensitization and stakeholder engagement both before and during new maternal vaccine introduction.

Our study results are similar to results from a recent study in Kenya on the decision-making process of introducing maternal vaccines (Otieno *et al*., 2021). Of note, our study only included county-level officials, while Otieno *et al*. (2021) included national-level officials as well. We also propose an adapted version of a new vaccine introduction framework (Levine *et al*., 2010) for use in the process of new maternal vaccine introduction. Unlike Otieno *et al*.’s (2021) findings, our county-level respondents understood that policies are set at the national level and did not report feeling left out of the process. The national officials included in Otieno *et al*.’s study highlighted the lack of a separate policy specific to maternal vaccines, as any new vaccine introduction would be based on the existing EPI-focused national policy guidelines. The county-level respondents in our study underscored this limitation of the national system by highlighting the importance of other national groups such as KOGS being involved in the development of a policy for any new maternal vaccine introductions. Like Otieno *et al*. (2021), our findings highlight the importance of stakeholder management at all levels, including the community level, to increase trust and improve uptake of new vaccines.

There have been several new vaccine introductions in low- and middle-income countries, and lessons learned from these experiences can be drawn upon to better understand information needs among maternal immunization policymakers and decision-makers. For example, there are several key parallels in the policy processes for the introduction of new maternal vaccines and for the introduction of the HPV vaccine, the most recently introduced vaccine in Kenya’s national immunization programme (World Health Organization, 2023b). Both maternal and HPV vaccines target specialized, beyond-infancy populations, requiring unique mobilization and delivery infrastructures, such as school-based vaccination programmes to target adolescents for HPV or maternal vaccine programmes based in antenatal care services to target pregnant women. The need to develop new programmes in resource-constrained settings with competing priorities has resulted in several low- and middle-income countries prioritizing the introduction of vaccines that are more easily integrated with existing EPI vaccine delivery systems—and, therefore, more cost-effective—over the introduction of the HPV vaccine (Gallagher *et al*., 2017; Guignard *et al*., 2019; Abdullahi *et al*., 2020). This idea was echoed by several participants in our study who discussed future maternal vaccine introduction, raising concerns over their cost-effectiveness, and highlighting the need to integrate them into existing antenatal care infrastructures instead of creating entirely new and costly parallel programmes.

Our results also show that policymakers value country-specific data, particularly on vaccine efficacy. However, importantly it is unlikely that it will be possible to generate context-specific efficacy data for every setting. To mitigate this challenge, it is vital to ensure that decision-makers are aware of what efficacy evidence is available and why it is relevant to their setting. Additionally, the importance placed by our study participants on integrating community sensitization and stakeholder engagement into the maternal vaccine policy adoption process has been reflected in multiple studies assessing the adoption of HPV vaccines. Ladner *et al*. (2016) evaluated lessons learned from HPV vaccine implementation programmes in 19 low- and middle-income countries and found that community misperceptions about the vaccine safety and efficacy were the largest obstacle to HPV vaccine uptake, more frequently cited than issues of audience reach, vaccine supply or logistics. Multiple studies on HPV vaccine adoption in Kenya found that the lack of consistent and effective stakeholder engagement with healthcare workers, religious leaders and community members contributed to HPV vaccine hesitancy and suboptimal uptake (Friedman *et al*., 2014; Vermandere *et al*., 2015; Karanja-Chege, 2022; Kolek *et al*., 2022). Njuguna *et al*. (2021) and Essoh *et al*. (2023) articulated how inadequate community education and information, combined with the fact that the HPV vaccine targets adolescent girls, has allowed for the spread of rumours that the HPV vaccine would render Kenyan girls infertile, negatively impacting vaccine acceptance. As upcoming maternal RSV and GBS vaccines have a sensitive target audience—pregnant women—they may be subject to similar misinformation and hesitancy, underscoring the importance of community sensitization and stakeholder engagement both before and during vaccine introduction, as recommended by our study participants. Addressing potential sources of vaccine hesitancy early in the policy implementation process may prove more effective than working to dispel misinformation after it has spread.

This study is not without limitations. Given the cross-sectional qualitative design, results are not generalizable to other settings. However, we believe understanding perspectives of local-level policymakers and decision-makers that may be involved in new maternal vaccine policy implementation is important.

## Conclusion

We are at an opportune time to understand what aspects ensure successful new maternal vaccine introductions and, in turn, increase acceptance and uptake. Given that maternal RSV and GBS are likely to become available over the next decade, having this context-specific insight may assist in the upcoming policy implementation and vaccine rollout process. It is also important to learn the perspectives of the county-level policy officials that will lead policy implementation once a national policy is put in place. Combatting vaccine hesitancy during the policy-implementation phase may assist in increased uptake and, therefore, increased population benefit of these new vaccines.

## Reflexivity statement

This manuscript is written by an all-female team with positions ranging from professor to doctoral student. Two authors are from Kenya. Five of the authors, including the first author and senior author, are women of colour.
